# What would ‘upscaling’ involve? A qualitative study of international variation in stroke rehabilitation

**DOI:** 10.1186/s12913-021-06293-8

**Published:** 2021-04-29

**Authors:** Kimberley Elizabeth Watkins, William Mark Magnus Levack, Farooq Azam Rathore, Elizabeth Jean Carleton Hay-Smith

**Affiliations:** 1 Department of Medicine, Rehabilitation Teaching and Research Unit, University of Otago, Mein St, Newtown, PO Box 7343, Wellington, 6242 New Zealand; 2 Department of Rehabilitation Medicine PNS Shifa Hospital DHA-II, Karachi, Pakistan

**Keywords:** Rehabilitation, Stroke rehabilitation, Physical and rehabilitation medicine, Developing countries, World Health Organization

## Abstract

**Background:**

Demand for stroke rehabilitation is expected to grow dramatically; with the estimated prevalence of stroke survivors rising to 70 million worldwide by 2030. The World Health Organization’s (WHO) report - *Rehabilitation 2030: A call for action* – has introduced the objective of ‘upscaling’ rehabilitation globally to meet demand. This research explored what upscaling stroke rehabilitation might mean for health professionals from countries at different stages of economic development.

**Methods:**

Qualitative descriptive study design using semi-structured interviews was employed. Purposively sampled, clinical leaders in stroke rehabilitation were recruited for interviews from low through to high-income countries.

**Results:**

Twelve rehabilitation professionals (medicine, physical therapy, occupational therapy, and speech and language therapy) from high (United States of America, Germany, United Kingdom, United Arab Emirates, New Zealand), upper-middle (Colombia and Turkey), lower-middle (Vietnam, Pakistan, Ghana), and low-income countries (Nepal and Sierra Leone) were interviewed. Upscaling was seen as a necessity. Successful scaling up will require initiatives addressing: political governance and managerial leadership, increasing knowledge and awareness of the value of rehabilitation, financial support, workforce developments, physical space and infrastructure, and the development of community services and reintegration.

**Conclusion:**

Although there have been many gains within the development of stroke rehabilitation internationally, further investment is required to ensure that this patient population group continues to receive the best quality services**.** For the WHO to be successful in implementing their objective to upscale rehabilitation, specific attention will need to be paid to political, professional, economic, and sociocultural issues at global and local levels.

**Supplementary Information:**

The online version contains supplementary material available at 10.1186/s12913-021-06293-8.

## Background

Disability caused by diseases and injuries is a growing challenge, especially in low and middle-income countries (LMIC). Between 1990 and 2010, the total global years lived with disability (YLD) from all causes increased from 583 million to 777 million, largely due to population growth and aging [1]. In 2015, the Global Burden of Disease Study identified that 74% of the total number of YLDs in the world arose from health conditions that would benefit from rehabilitation [2]. As a result of raised awareness for the growing international need for rehabilitation, in 2017 the World Health Organization launched *Rehabilitation 2030 -* “a call for action to scale up rehabilitation so that countries can be prepared to address the evolving needs of populations up to 2030.” ([3], p.12) What might be required to scale up rehabilitation successfully will differ, however, between countries depending on each country’s economic, political, cultural, and social context. Few data are available about clinician views of what upscaling rehabilitation means for them and the nature of upscaling they think is needed within the services they currently provide.

The main aim of this preliminary qualitative study was to investigate the views and experiences of experts in stroke rehabilitation from 12 different countries regarding what upscaling rehabilitation might mean in their local context. Qualitative research is important for international health strategies such as *Rehabilitation 2030* as it “helps policy-makers and program managers to make decisions about how to adapt a given WHO guideline and how to prioritize specific recommendations for implementation.” ([4], p.79) We chose to focus on stroke rehabilitation, as stroke is the second leading cause of death and the third leading cause of disability worldwide [5].

Our research team chose to interview clinicians working on the front line of stroke rehabilitation. The insights from this group may be of interest to other clinicians in similar contexts, and usefully add to the views of others (such as policy-makers and program managers) to provide a picture of what may be required when ‘upscaling’ services.

## Methods

### Research team and study design

Our research team comprised of two women (KW and EJCH-S) and two men (WL and FR) based in New Zealand (WL and EJCH-S), Italy (KW), and Pakistan (FR). Two of our team were experienced qualitative researchers with PhDs (WL and EJCH-S), two were registered physiotherapists (KW and WL), and one was a physician and rehabilitation researcher (FR). This research employed qualitative descriptive analysis methods [6] situated within a constructionist epistemology. One of our research team (FR) also acted as a study participant from a lower middle-income country, contributing to the research’s emic stance. Ethics approval was provided by the University of Otago Human Ethics Committee (Health) prior to any participant recruitment (18/086).

### Participant recruitment

To be included in the study, participants needed to be currently working in stroke rehabilitation; have at least 5 years of clinical experience; be a physician, rehabilitation nurse, physical therapist/physiotherapist (PT), occupational therapist (OT), or speech language therapist (SLT) in a leadership role in their rehabilitation team; have sufficient proficiency in English to describe their clinical practice in an interview; and have internet or phone access. We identified potential participants through our international networks, including Cochrane Rehabilitation, and by word of mouth. We used purposeful sampling to select potential participants from a range of low, lower-middle, upper-middle, and high-income countries, using the World Bank Atlas method to classify countries by gross national income [7]. We aimed to recruit 8–12 participants, as this seemed likely to yield sufficient detailed data for the purposes of qualitative descriptive analysis [8]. All 12 participants were contacted via email and provided written consent. The participants were given the option to be identifiable or anonymous in the write up of this report.

### Data collection

We used semi-structured interviews conducted by videoconference or phone to collect data and provided all participants with a copy of the main questions prior to their interview. The interview guide developed for this study is provided as Additional file 1. One researcher (KW) conducted all the interviews, with supervisory input from the rest of the team. Two participants were interviewed on two occasions to accommodate their availability and time constraints. The interviews ranged from 60 to 90 min. All interviews were audio-recorded and then transcribed verbatim. We offered participants an opportunity to edit or amend the transcripts of their interviews.

### Data analysis

We used inductive content analysis to examine the interview transcripts [9, 10]. NVivo software (QRS International) was used to help manage the interview data and data coding [11]. Each transcript was read and re-read, incorporating the findings from additional interviews as the study progressed [9, 10]. One researcher (KW) undertook initial coding (open coding) on a line-by-line basis. In subsequent analysis, we explored the relationships between codes to identify overarching concepts (coding tree available on request). To strengthen the credibility and trustworthiness of the analysis [10], two other researchers (WL and EJCH-S) each independently completed an analysis of 30% of the interview transcripts. These two researchers then compared and debated their findings with the first researcher, along with reflections on the data collection, analysis, and study process in weekly team meetings. In addition, one of the research team (FR) provided feedback on the codes and themes from the perspective of someone working in stroke rehabilitation in a lower middle- income country. We also sent a draft of our initial analysis to each participant for comments or corrections. Feedback from three participants was provided and incorporated into the final analysis. The results below are presented with a number of extracts to illustrate key points.

## Results

Twelve experts in stroke rehabilitation were purposefully recruited from several regions, professions, and work settings (see Table 1). Three participants wished to remain anonymous and are referred to by their interview number in place of their names. Five participants were from high-income countries (USA, Germany, United Kingdom (UK), United Arab Emirates, New Zealand), two from upper-middle income countries (Turkey, Colombia), three from lower-middle income countries (Pakistan, Vietnam, Ghana), and two from low-income countries (Nepal, Sierra Leone). Participants worked across the continuum of stroke rehabilitation services from acute care, inpatient rehabilitation, outpatient and community rehabilitation. Six further potential participants (Canada, Nigeria, Ethiopia, Bangladesh, China, Malaysia) expressed interest but did not take part, primarily because we were unable to schedule interviews in a timely manner due to difficulties with communication and busy work schedules.

**Table 1:** Characteristics of study participants

Participant	Profession	World Region and income of country (GNI per capita)^a^	Years of experience	Local work setting
Dr. David Alexander	Neurologist	USA (North America) High-income: $62, 850	35	Inpatient and outpatient
Gabriele Eckhardt	PT	Germany (Europe) High income: $47, 180	35	Outpatient rehabilitation center
Participant 7	PT	United Kingdom (Europe-UK) High income: $41, 340	5–10^b^	Inpatient and acute hospital setting
Participant 12	SLT	United Arab Emirates (Middle East) High-income: $41, 010	5–10^b^	Rehabilitation center
Participant 2	OT	New Zealand (East Asia and Pacific) High-income: $40, 820	10–15^b^	Inpatient hospital setting
Dr. Ali Yavuz Karahan	Physiatrist (PRM physician)	Turkey (Europe) Upper middle- income: $10, 380	10	University hospital- inpatient and outpatient setting
Dr. Vanessa Seijas	PRM Physician	Colombia (South America) Upper middle-income: $6190	5	Inpatient and outpatient setting
Bui Thi Huong	PT and OT	Vietnam (East Asia) Lower middle-income: $2, 400	9	Inpatient and outpatient rehabilitation hospital setting
Dr. Abena Tannor	PRM physician	Ghana (Sub-Saharan Africa) Lower middle-income: $2130	8	Inpatient and outpatient hospital setting
Dr. Farooq Rathore	PRM physician	Pakistan (South Asia) Lower middle-income: $1, 580	10	Inpatient and outpatient setting
Roshan Kumar Bishwakarma	PT	Nepal (South Asia) Low-income: $960	7	Inpatient and outpatient rehabilitation center
Ismalia Kebbie	PT	Sierra Leone (Sub-Saharan Africa) Low income: $500	9	Inpatient and outpatient hospital setting

*Abbreviations*: *GNI* Gross National Income, *OT* occupational therapy, *PRM* Physical and rehabilitation medicine, *PT* physiotherapy/physical therapy, *SLT* Speech and language therapy, *SW* Social Worker

^a^GNI per capita are from the 2017 or 2018 fiscal year dependent on updated information, presented in US dollars, and reported based on the World Bank Atlas method for calculating GNI, plus for categorizing countries as low, lower-middle, upper-middle, and high income [7, 12]

^b^Within the data a range of years of work experience has been included to protect participant anonymity

Reference [7, 12]

### Overview of study findings

The participants provided various descriptions of what might constitute upscaling of stroke rehabilitation, with no single description prevailing. What might be needed to upscale stroke rehabilitation differed across and within countries. Despite these differences, all participants viewed the upscaling of stroke rehabilitation as positive and necessary; essential for helping stroke survivors return to their community and meaningful activities. The core concepts of upscaling stroke rehabilitation were to improve the quality and reach of services, and to ensure everyone who would benefit from rehabilitation had access.

Participants in the high-income countries tended to describe upscaling in terms of improving the quality and accessibility of stroke rehabilitation services to all people across their countries. They described access to stroke services as inequitable in their countries, with variation occurring due to cultural, geographic, and economic reasons. Upscaling would allow more patients to receive the individual rehabilitation they required.

The participants in the upper middle-income countries reported that upscaling would require the recruitment of more rehabilitation professionals with specialist training in stroke rehabilitation, as well as greater development of dedicated multidisciplinary teams. They also reported that access to services needed to improve in rural areas. The participants in the lower middle and low-income countries viewed upscaling as fundamentally necessary because it was currently so limited by workforce and infrastructure constraints. In Sierra Leone, for example, physiotherapists led all stroke rehabilitation because other health professionals, including physicians, had no specialist training in rehabilitation.

*It is very important because rehabilitation, especially in third world countries, which is not taken as a priority but is sometimes very needed. We must just not focus our attention on keeping people alive, but rather adding some value or quality to the life that we are saving. So, investing in rehabilitation or scaling up rehabilitation is one of the best ways we can still make use of the human resources that we have in the country. (PT, Sierra Leone)*

The participants discussed several areas of consideration for upscaling to be successful. These areas included: politics, leadership, and governance; increasing knowledge and awareness of stroke and stroke rehabilitation; financial support; workforce development; physical space and infrastructure for rehabilitation; and increasing the emphasis on community services and community reintegration. We discuss each of these in more detail below, with further examples from the interview transcripts provided in Table 2.

**Table 2:** The main themes with an additional supplementary supporting quote

Theme	Supporting quote
Politics, leadership, and governance	None of this can be implemented if you don’t involve the government. This is how it works, at least in the lower-middle-income countries that not everything can be done by the NGOs [non-governmental organizations] or by public support. It has to come from the administration or the country’s head of the government. (PRM physician, Pakistan)
Increasing knowledge and awareness of rehabilitation	I think it would be good for professionals … to be an expert in that area increasing awareness in the public, within families, and education allowing to integrate the patients more easily into the communities and within their home environments. I mean just providing a better quality of life for them (SLT, United Arab Emirates)
Financing stroke rehabilitation	Changing the insurance options for the patient and not only government funding can positively affect rehabilitation (PRM physician, Turkey)
Workforce development	Having a case manager or caregiver, somebody who made sure the patient connected with their doctors and their therapists and gave simple advice that they need, things like that. That would be useful. It’s not really hard to find it. We don’t have the mechanism right now to have somebody paid to do that. (Neurologist, USA)
Physical resources and infrastructure	Then also the infrastructure. It doesn’t have to be high tech equipment being donated, but we need to be able to adapt because we can’t constantly rely on developing countries to provide everything. (PRM physician, Ghana).
Community services and reintegration	You can improve quality of life for the patient and help the family if the patient can come back (to) their work, they can get money, or they can (be) less dependent in ADL’s [Activities of daily living] (PT/OT, Vietnam)

*OT* occupational therapy, *PRM* Physical and rehabilitation medicine, *PT* physiotherapy/physical therapy, *SLT* Speech and language therapy

Note: Transcription conventions are as follows: round parentheses () are used when indicating an added explanation; square parentheses [] are added to explain an abbreviation; and ellipses … indicate the removal of a word or words without altering the meaning

### Politics, leadership, and governance

The participants considered political will to be essential for successful upscaling of stroke rehabilitation. Without governmental support, upscaling was considered nearly impossible. Assistance was needed from all managerial levels, and institutional support was required to make changes within the healthcare systems. It was crucial for healthcare systems and leaders to make stroke rehabilitation a priority area.

*Even if it’s not the government, management of these hospitals should be able to get them to appreciate it - because eventually, they would have to spearhead it. We are physicians, we can talk, but we can’t decide to go take a room by force and start a rehab unit. So, it should be initiated from the management of hospitals and also from the government. (PRM physician, Ghana)*

### Increasing knowledge and awareness

The participants believed that government officials, health services managers, health funders, and community members needed to understand and appreciate the value of stroke rehabilitation before the upscaling of services could be truly successful. Government officials needed more knowledge and awareness of stroke rehabilitation and its health and economic value, so that they could lead the changes needed to health policy and infrastructure. However, other health professionals working in acute medicine or primary care also needed to appreciate (or, in some of the LMIC’s, be educated in) the value of rehabilitation to refer to those services so that post-discharge care included necessary rehabilitation input.

Furthermore, sociocultural factors and health education influenced the knowledge of the causes of stroke among the general population and therefore, directly influenced patients and families choosing to access rehabilitation services in a timely manner. In Ghana and Sierra Leone, some people attributed stroke to a curse or spiritual cause; this resulted in family members not accessing rehabilitation services at all, or only accessing services after impairments had become significant health problems (e.g., spasticity turning into irreversible contractures). In the United Arab Emirates some patients and families, who had a strong belief that divine intervention would heal them, did not easily accept the rehabilitation interventions offered. Therefore, education of the public was required in some countries for patients and families to engage with rehabilitation services and access them promptly when needed.

*It [uptake of rehabilitation] is low because there is a lot of emphasis on the spiritual aspect of the disease. So, a lot of them [patients/families] believe it’s either a curse or a spiritual cause, or it can be cured with herbal preparations. So, a lot of them after being discharged from the acute stroke inpatient ward are going to see a traditionalist, herbalist, or spiritualist to help. Then they are coming back weeks or months later with complications. (PRM physician, Ghana)*

### Financial support

All participants reported that financial investment was required to scale up stroke rehabilitation. In some of the lower-income countries, funding was required to establish and expand the coverage of rehabilitation services. However, even in high-income countries the participants reported that the upscaling of stroke rehabilitation would require more than just the reorganization of existing services and resources.

*It’s not very helpful to be saying … we want you to upscale your rehab – for that to be said without any extra resources of finance attached to it. Otherwise, they can say it until they are blue in the face, but it is never going to happen. (OT, New Zealand)*

### Workforce developments

When investing in the development of stroke services, the participants all emphasized the need to invest in the healthcare workforce first – before investing in buildings, equipment, or other physical resources for rehabilitation. The participants argued that their governments and health service leaders needed to have a long-term strategy to recruit and cultivate these professionals within their countries. In the high-income countries, the participants discussed that this might also mean establishing new roles to support the healthcare system as a whole. Suggested roles were those with a focus on stroke service development or evidence-based practice implementation, case management, rehabilitation nurse specialist, and new or expanded ‘lay’ caregiver roles including patient advocate, educator or navigator.

*That is more of a big problem for the future because the statistics say – the forecast says in 2030, we will have 70 million stroke survivors in the world. So, we will have a lot more people in our rehab center, and we can’t take care of them if we don’t have the basic (staff) for working. That’s the biggest problem … it’s like running with closed eyes in front of a wall. (PT, Germany)*

In addition, participants in the middle and low-income countries stated that there needed to be more rehabilitation professionals recruited, especially in rural areas, to improve access to services. These countries needed more of the specialist rehabilitation professional groups represented, who were often not available at all (e.g. physical and rehabilitation medicine physicians, occupational therapists, and speech and language therapists) and up-skilling of all rehabilitation professionals in stroke rehabilitation, the application of EBP, and multidisciplinary team approaches.

*What we want first of all is to provide the right human resources. That’s very key those are the services providers. We want to ensure the right human resources are available. (PT, Sierra Leone)*

Access to specialist training in rehabilitation was another limitation in middle and lower-income countries. Often specialist training was only available if people traveled to higher-income countries, which limited how many people could receive this training. Many participants thought a more efficient way of training was to invite a trainer or a trained professional from a high-income country.

*We can’t send more than twenty staff to foreign countries, but we can teach or educate more than 50 people at the same center, which is conducted in Turkey. So, I think first we need a real education [in rehabilitation] in Turkey. (PRM physician, Turkey)*

### Physical space and infrastructure

All participants believed that they needed further access to physical resources for rehabilitation if upscaling were to be successful. However, differences were apparent in what resources were required depending on the local context. Participants in the upper-middle and high-income countries emphasized the need for funding of additional equipment or technology to support assessment, diagnosis, and treatment, to increase the number of rehabilitation beds available. Participants in lower-middle and low-income countries experienced more challenges with inadequate space for rehabilitation.

*My personal challenge is that I don’t have adequate space. I mean if you see my department on any busy day, it would resemble more of a fish market than a rehab department (PRM physician, Pakistan).*

In some countries, patients and families without health insurance or the ability to pay privately had limited access to diagnostic tests as well as primary rehabilitation interventions. This situation directly influenced their opportunity to receive evidence-informed rehabilitation.

### Community services and reintegration

The participants from all countries spoke of the importance of rehabilitation services moving into communities (post-discharge, but also treated in the community without hospital admission) and of developing support for community reintegration after stroke, with a particular emphasis on helping patients return to work.

*They (patients) lose their jobs, they cannot go back to their normal activities, and these are people that are very much useful in society (PT, Sierra Leone)*

Community-based rehabilitation and returning to work was also viewed as a way of tackling some of the disability-associated stigma, especially in the LMIC’s.

*Returning these patients to work as useful, earning members of society could help reduce the stigma of disability in Pakistan (PRM physician, Pakistan).*

However, gaps in services after discharge from the hospital were apparent even in high-income countries, with variable coverage for community-based rehabilitation. The participant from the UK used the phrase ‘postcode lottery’ to refer to the arbitrary influence of where patients happened to live on the quality of community rehabilitation they received.

Participants from the middle and low-income countries said community services were sparse (to non-existent), which affected the patients’ opportunity for reintegration into their communities. Investing in community services in the rural areas was seen as an essential area for upscaling rehabilitation. Some wanted specialist members of the multidisciplinary team in the rural areas so that patients did not have to travel to the larger cities to receive the same level of care and rehabilitation. Telerehabilitation was suggested by one participant as a way to improve rural service access, and this was being tested by supporting general practitioners in rural towns and hospitals by connecting them with a specialist doctor in a larger centre.

*Only if the government understood that we need the services of rehabilitation in the primary care level. For example, in the tiny hospital that I talked about which is in the distant areas, they should have some rehabilitation care because if the patient has to come to the big city to have it, it is not good (PRM physician, Colombia).*

## Discussion

This study provides insights into what health professionals working in stroke rehabilitation in a variety of international settings think might be required to achieve the WHO’s *Rehabilitation 2030: A call for action* [3]. While our study focuses primarily on stroke rehabilitation, we suggest that the core themes highlighted here are likely to be relevant to other areas of rehabilitation internationally. Our findings suggest that while all countries need and will benefit from upscaling rehabilitation, how this objective is achieved will differ from country to country. All participants identified unmet rehabilitation needs regardless of country income, although participants from LMIC identified a greater lack of infrastructure and workforce to meet these needs and also (in some settings) cultural understandings of stroke that acted to reduce patient engagement with rehabilitation.

Much of the prior research on rehabilitation in LMIC has focused on highlighting or quantifying the lack of rehabilitation services, especially in rural and economically poorer regions, and this impact on poorer health outcomes [13–19]. Even with the Global Stroke Services Guideline and Action Plan providing recommendations for minimal, essential, and advanced levels of service provision [20], our study indicates some challenges to achieving this consistent with the challenges identified by others for implementing rehabilitation services in LMIC settings. For instance, Khan et al. [21] reported that multiple LMICs (Nigeria, Madagascar, Pakistan, and Mongolia) identified common challenges to implementing the WHO Global Disability Action plan. These included the need for rehabilitation leadership at governance, policy, and management level; investment in rehabilitation infrastructure; an emphasis on specialist rehabilitation training of health professionals; a focus on the development of community-based rehabilitation; and the need for raising knowledge and awareness [21]. More recently, Morris et al. [22] considered the challenges of implementing the WHO 2030 Rehabilitation goals in South Africa using the WHO health system building block framework. They found a need for increases in rehabilitation workforce and financing, more access to medical technologies, and more leadership and support from those in governance roles [22].

Like other areas of healthcare [23], upscaling of rehabilitation is limited by a global shortage of specialist trained health professionals. Not only is there a need for the right professionals and strong multidisciplinary teams, but also a need for equitable and affordable access to these professionals and services both across and within countries. In this study, even participants from high-income countries believed that access to specialist rehabilitation services was inequitable – with access limited by funding, geographical location, and other arbitrary factors.

We suggest two topics worthy of further research. First, to ask those in governance roles (e.g. ‘helicopter’ rather than ‘coal-face’ views of upscaling rehabilitation) to consider what is needed to meet ‘evolving needs’ [3] in stroke rehabilitation (e.g. the increasing epidemic of multiple co-morbidities in the aging population that will increase the complexity of care needs). Second, the influence of cultural, social, and religious views on rehabilitation uptake [24–28]. Our participants experienced difficulties providing evidence-based rehabilitation for those with low health literacy, especially when this conflicted with spiritual beliefs or cultural norms that valued traditional therapies and healers. More research is needed exploring how to increase knowledge and raise awareness about stroke (e.g. exploring cross-cultural differences in how stroke is conceptualized) and perceptions about the value of stroke rehabilitation.

### Study limitations

We interviewed one health professional in each country: different health professionals from different backgrounds and regions might well have emphasized other issues when discussing upscaling stroke rehabilitation in their countries. We were also limited to interviews in English, which restricted the type of information we were able to gather and from whom. There may be cultural factors related to upscaling stroke rehabilitation that we failed to identify.

## Conclusion

Rehabilitation is now a core part of the definition of Universal Health Coverage [29] and therefore access to stroke rehabilitation should be considered a human right. Global sharing of knowledge, training, and resources is essential, but successful upscaling of rehabilitation will depend on local solutions driven by local people. Successful implementation of the WHO’s plan to scale up rehabilitation will certainly require political, professional, economic, and sociocultural issues are addressed at global and local level.

## Supplementary Information

**Additional file 1.** Overview of the interview guide.

## Data Availability

Data generated or analyzed during this study are included in this published article, additional data can be requested from the corresponding author if required.

## Abbreviations

LMIC: Low and middle-income countries
YLD: Years lived with disability
WHO: World Health Organization
PRM physician: Physical and rehabilitation medicine physician
PT: Physiotherapist/Physical therapist
OT: Occupational therapist
SLT): Speech and language therapist
