# Supplementary material for: What would ‘upscaling’ involve? A qualitative study of international variation in stroke rehabilitation
Source: BMC Health Serv Res. 2021 Apr 29;21:399. doi: 10.1186/s12913-021-06293-8 (PMC8082958; doi:10.1186/s12913-021-06293-8)
Supplement: Supplementary file 1 — Additional file 1. Overview of the interview guide. [file 12913_2021_6293_MOESM1_ESM.docx]

**Additional File 1: Overview of interview guide**

1. **Would you please tell me about what stroke rehabilitation in your current workplace usually involves?**

| What does stroke rehabilitation look like in your particular work setting? |
| --- |
| In usual practice, what is actually provided to stroke patients to address problems with mobility/communication/activities of daily living? |
| What do you think are the biggest influences over clinical decision-making in your stroke rehabilitation service? – (Compared to what you would ideally like to deliver?) |

1. **Would you please tell me about how easy or hard it is for your team to apply evidence-based practice in the stroke rehabilitation services that you can usual offer patients?**

| How do you find out about developments in evidence-based practice in stroke rehabilitation? |
| --- |
| What do you find are the main barriers to your team implementing more evidence-based therapy in their usual practice? (i.e. time, resources, caseload, practicality of work setting) |
| What do you think most helps facilitate your or your team’s implementation evidence-base practice in your usual clinical practice? |

1. **The World Health Organization has recently introduces its Rehabilitation Strategy to 2030, in which it talks about the need to ‘upscale’ rehabilitation worldwide. What would ‘upscaling’ stroke rehabilitation mean in your region?**

| What does ‘upscaling’ rehabilitation mean to you and your team’s clinical practice? |
| --- |
| What would be needed to ‘upscale’ rehabilitation in your region? |
| If successful, what do you think the consequences of upscaling stroke rehabilitation in your region could be? |
| Do you have any views on what the consequences of ‘upscaling rehabilitation’ might be for evidence-based practice in stroke rehabilitation? |
